# The Extent of the Crack on Artificial Simulation Models with CBCT and Periapical Radiography

**DOI:** 10.1371/journal.pone.0169150

**Published:** 2017-01-04

**Authors:** Shuang Wang, Yiran Xu, Zhengyan Shen, Lijun Wang, Feng Qiao, Xu Zhang, Minghua Li, Ligeng Wu

**Affiliations:** 1 Department of Endodontics, School of Stomatology, Tianjin Medical University, Tianjin, China; 2 Department of Endodontics, Stomatological Hospital of Yantai, Shandong, China; 3 Department of Radiology, School of Stomatology, Tianjin Medical University, Tianjin, China; 4 Department of Maxillofacial Surgery, School of Stomatology, Tianjin Medical University, Tianjin, China; 5 BYBO Dental Group, Beijing, China; University of Notre Dame, UNITED STATES

## Abstract

**Background:**

The aim of this study was to investigate the extent of the crack of a cracked tooth on an artificial simulation model with Periapical Radiography (PR) and cone beam computed tomography (CBCT) *in vitro*, providing the basis for early diagnosis and an appropriate treatment plan.

**Methods:**

Forty-four teeth with different extents of artificial cracks, created by exposure to liquid nitrogen after hot water at 100°C, were collected. They were subjected to PR and CBCT. Micro-computed tomography (micro-CT) examination, regarded as a relatively more accurate measurement than others, was used to measure and record the crack depth. Three observers, an endodontic graduate student, an experienced endodontist, and an experienced radiologist, examined the PR and CBCT results independently, and the presence or absence of cracks with PR and CBCT were respectively recorded. The external consistency ICC with 95% confidence interval (95% CI) was used to analyze the consistency among the graduate student, endodontist, and radiologist; ROC curves were used for the analysis of diagnostic performance of both radiographic modalities for tooth cracks with crack depth.

**Results:**

For the interpretation of the PR results, there were statistically significant differences among the three different observers (*P <* 0.001), and the interpretation of the CBCT results (*P <* 0.001). In the group of results read by the graduate student, the sensitivity of diagnosis with CBCT and PR was 77.27% and 22.73%, respectively (*P* < 0.001). In the group of results read by the endodontist, the sensitivity of diagnosis with CBCT and PR was 81.81% and 8.19%, respectively (*P* < 0.001). In the group of results read by the radiologist, the sensitivity of diagnosis with CBCT and PR was 88.64% and 11.36%, respectively (*P* < 0.001). As for CBCT diagnosis, the critical value for the graduate, endodontist, and radiologist was 3.20 mm, 2.06 mm, and 1.24 mm, respectively. For the PR diagnosis, the critical value for the graduate, endodontist, and radiologist was 6.12 mm, 6.94 mm, and 6.94 mm, respectively.

**Conclusions:**

Within the limitations of this study, on an artificial simulation model of cracked teeth for early diagnosis, we recommend that it would be better for a cracked tooth to be diagnosed by a radiologist with CBCT than PR, CBCT with a minimum depth of 1.24 mm.

## Introduction

Tooth cracks have become the third largest cause of tooth loss after dental caries and periodontal disease [1]. Early enamel cracks have no obvious symptoms, and patients often fail to see a dentist. Most patients with cracks who do see a dentist do so whilst suffering because of pulpitis and periapical periodontitis, or even root fracture [2]. This creates a great challenge for designing an appropriate treatment plan and assessing the long-term prognosis for cracked teeth [3]. Kim [4] studied 72 cracked teeth, and different treatment plans were undertaken based on their differing clinical symptoms. Tooth cracks exhibit these different clinical symptoms as a function of depth; when the crack is only in the enamel or superficial dentin, the teeth may be asymptomatic, or exhibit only dentin hypersensitivity to cold, sweet, and sour stimuli. If there is dental pulpitis or periapical periodontitis, however, the crack may have reached to the deep dentin layers or invaded the pulp cavity. Michaelson [5] reported 3 cases of cracked teeth. In early treatment, the crack was visible, but was not assessed for its range or depth. Additionally, no measures were taken to interfere with crack development. Though the depth of the crack in these cases was within the clinical treatment limit, a good therapeutic effect was still achieved. Nonetheless, early diagnosis and treatment can save the vital pulp of a cracked tooth where positive outcomes would otherwise be difficult [6,7].

Therefore, early intervention for cracked teeth is more likely to produce a better long-term prognosis, which can obviate the need to repair cracks after root canal therapy, as mentioned in the above literature; it can also avoid tooth pain, or the need for tooth extraction after installation of a crown prosthesis. However, if cracked teeth have advanced to developing pulpitis or periapical periodontitis, the prognosis is poorer than if early intervention therapy is pursued. The current consensus amongst researchers is that the early diagnosis of a cracked tooth is the key factor in determining whether the treatment plan is successful and prognosis is positive [8]. As diagnosis and treatment require precise information regarding the location and depth of the crack, it is a long-term problem for endodontists to obtain this information.

There are many methods for the diagnosis of cracked teeth. When the crack extends to the mesial and/or distal marginal ridges, it can be easily diagnosed through macroscopic observation, iodine staining, transillumination methods, microscope observation, and other methods in combination with clinical manifestations. In clinical settings, however, the commonly used technique of Periapical Radiography (PR) cannot diagnose tooth cracks, especially when the crack is extending mesial to distal, or the crack is parallel to the tooth length axis, or less than a certain angle [9]. Reports on the auxiliary diagnosis of vertical and cross root fracture indicate that Cone Beam Computed Tomography (CBCT) can clearly show crack depth, scope, and contours [10–13]. Nonetheless, there are no reports on the diagnosis of cracked teeth with CBCT [8,14–16].

In view of the above, most clinical endodontists think that CBCT cannot be used for the auxiliary diagnosis of a cracked tooth. In this study, a simulated artificial tooth-crack model was created to examine whether crack depth affected the ability of PR and CBCT to diagnose tooth cracks. This may provide certain theoretical bases for early treatment plans, and the evaluation of long-term prognosis.

## Materials and Methods

### Objectives

We collected 60 intact teeth extracted with minimally invasive extraction due to periodontic or orthodontic reasons at the Stomatological Hospital of Tianjin Medical University. The inclusion criteria were no visual evidence of external cracks, craze lines, or fracture with the naked eye following extraction. The exclusion criteria were the presence of dental caries, root absorption, severe abrasion, wedge-shaped defects, and horizontal or vertical root fractures. The patients were informed and signed an informed consent document for clinical research. This research was approved by the ethics committee of the Stomatological Hospital of Tianjin Medical University (TMUSHhMEC2014070).

### Establishment of an *in vitro* artificial simulation cracked tooth model

All 60 freshly extracted teeth were placed in a 1% sodium hypochlorite solution for 24 h for the removal of soft tissue and calculus. They were then soaked in 0.9% saline solution no more than one month prior to use. Before establishing an *in vitro* artificial cracked tooth model, the 60 extracted teeth were screened again and determined to have no cracks, craze lines, or fracture with a surgical operating microscope (S7/OPMI PROergo; Carl Zeiss, Oberkochen, Germany) under 17× magnification. Thereafter, they were soaked in 100°C hot water for 10 min, and were then quickly transferred into liquid nitrogen (-130°C) for 1 min. Among the 60 teeth, 44 were selected as having positive surface cracks using the same microscope under the same magnification; the remaining 16 teeth without a crack were excluded.

### Image acquisition and estimation of PR and CBCT

All 44 cracked teeth were randomly embedded in four trays, which were identical in size and filled with wax to represent periodontal tissue. Then, PR (Sirona, Germany) and CBCT (Kavo 3D exam, Germany) readings were taken. The parameters of the PR were 70 kV, 70 mA, 0.07 s, with the angle for the pitch tube perpendicular to the tooth length axis and film, and the film parallel to the tooth length axis. The parameters of the CBCT were 120 kV, 5 mA, the matrix size was 640 × 640, Field-of-View = 16 × 13 cm, and the pixel size was 0.25 mm × 0.25 mm.

**Fig 1:**
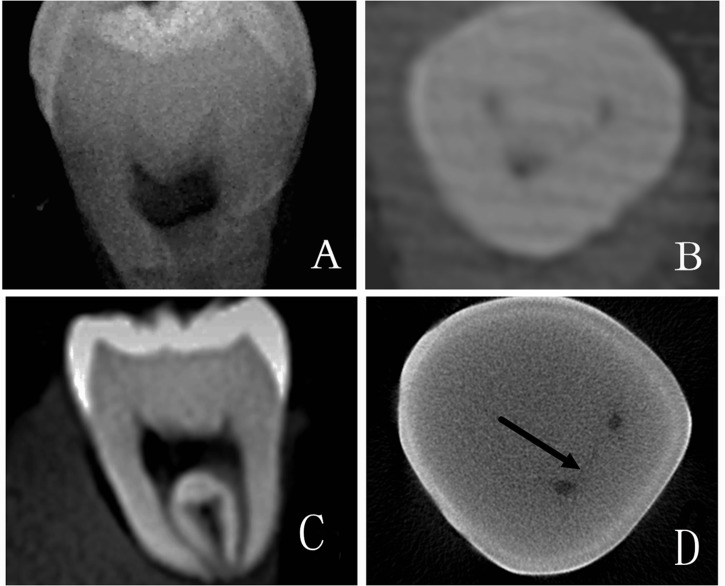
Group A, in which neither PR nor CBCT could detect cracks. A PR image of the sample is shown in panel A. Different cross-sections of CBCT are displayed in panels B and C. No cracks were detected in those images. A Micro-CT scanning image is shown in panel D, proving the existence of a crack. The crack is indicated by the arrow in the picture.

**Fig 2:**
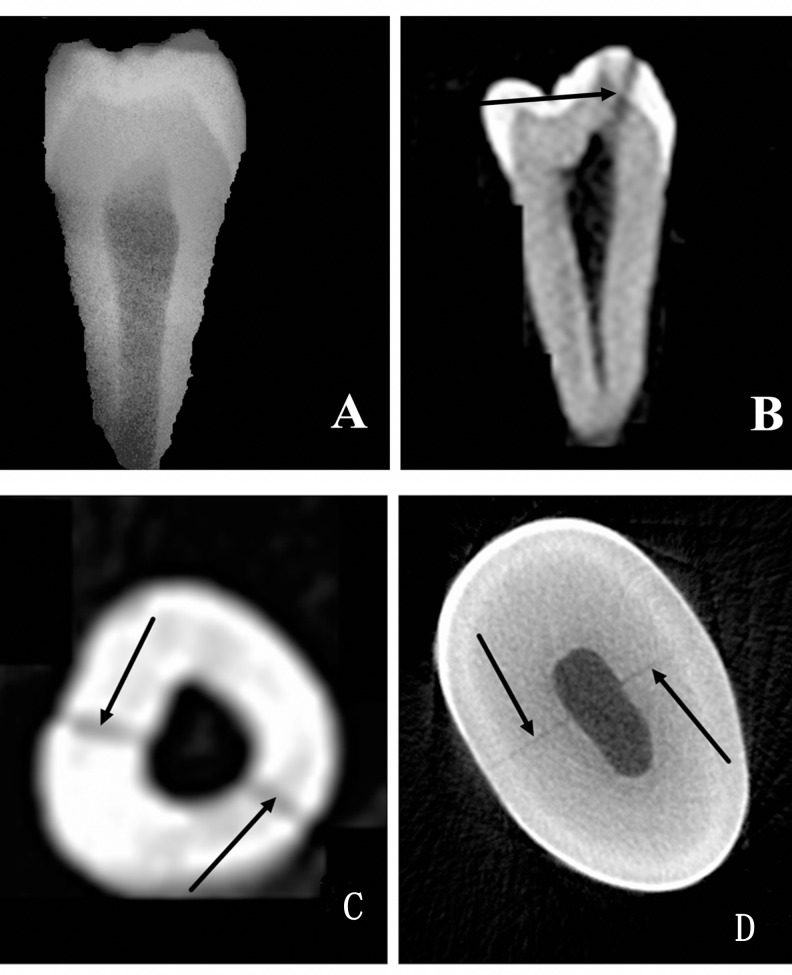
Group B, where only CBCT could detect the crack. A PR image of the premolar tooth is shown in panel A without any detected cracks. With CBCT, a crack was visible in different cross-sections as indicated by an arrow. A Micro-CT scanning image is shown in panel D, with the crack indicated by arrows.

**Fig 3:**
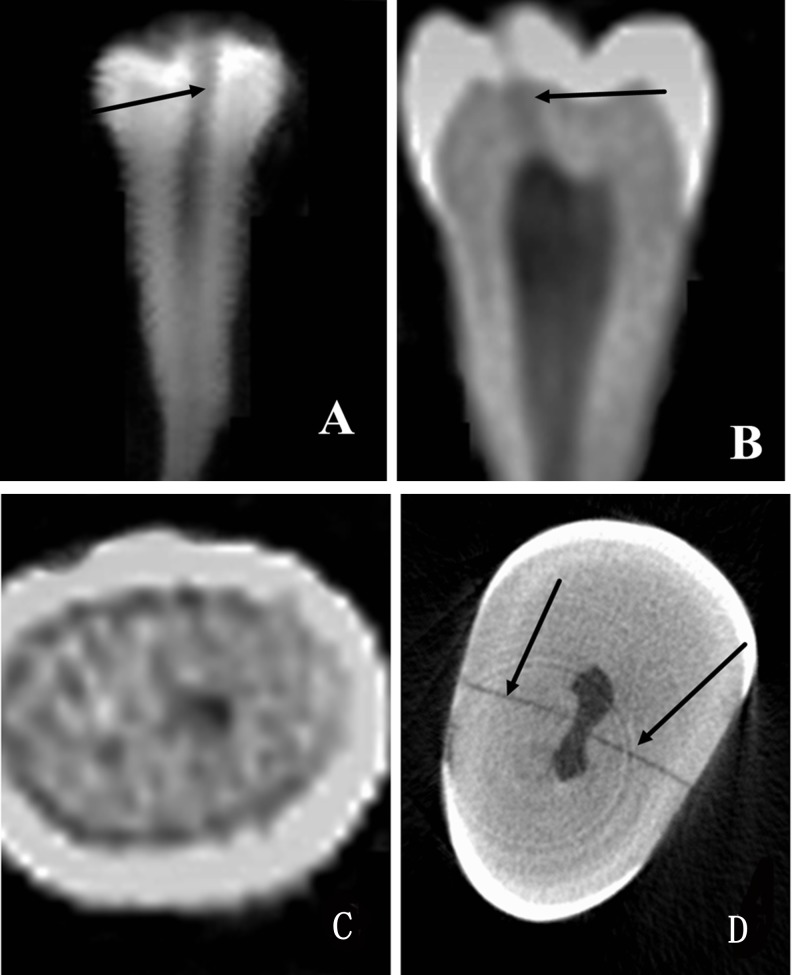
Group C, where both CBCT and PR can detect the crack. **An image of the crack in a premolar tooth is shown**. The crack was detected by PR (*A*) (arrow). The crack was detected by CBCT in the sagittal plane (*B*) (arrow). No crack was detected by CBCT in the same sample in the horizontal plane (*C*). The crack was detected by Micro-CT in the horizontal plane (*D*) (arrow).

Three observers, an endodontic graduate student, an experienced endodontist, and an experienced radiologist examined the PR and CBCT results independently, and the presence or absence of cracks with PR and CBCT were recorded respectively. The positive and negative results of each observer were separated into 3 groups: neither CBCT nor PR could detect the crack in group A (**Fig 1**), only CBCT could detect the crack in group B (**Fig 2**), and both CBCT and PR could detect the crack in group C (**Fig 3**).

In order to ensure the quality of the radiograph, all the processes were completed by one operator. Image data acquisition was completed and the results stored in a database. CBCT images were analyzed in the Vision Q (KaVo eXam Vision) software package.

### Micro-CT analysis

To evaluate the accurate crack depth of all 44 teeth, micro-CT was undertaken using a Skyscan1174 compact Micro-CT system (Skyscan 1174 v2, SkyScan N.V., Kontich Germany). Images were scanned at a resolution of 13 μm, tube voltage of 50 kv, tube current of 800 μA and exposure time of 1700 ms in each of the 360 rotational steps. The images consisted of hundreds of slices with a voxel size of 26 μm in all three axes. Two-dimensional images were used to generate 3D reconstructions using the visualization software NRecon (http://bruker-microct.com/products/1174.htm).

**Fig 4:**
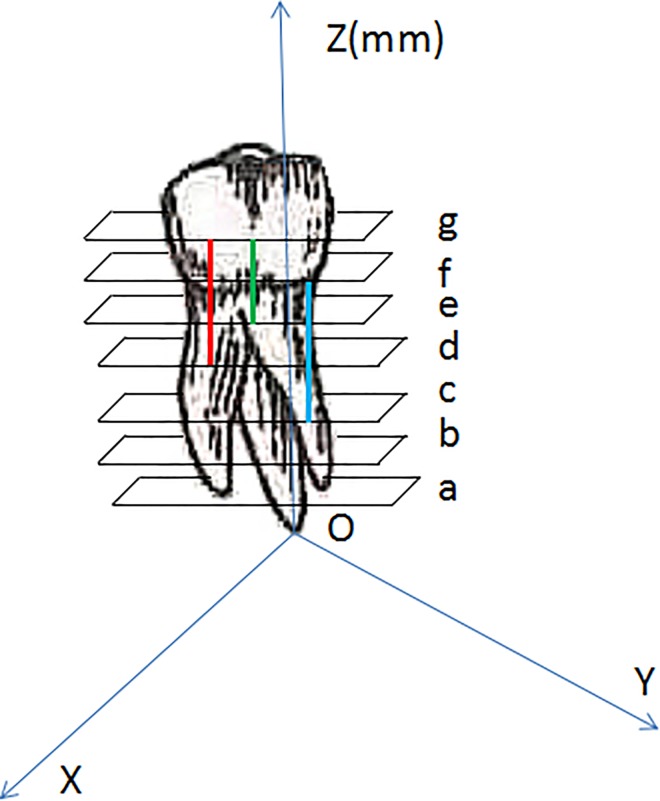
Schematic diagram showing how to calculate the crack depth with micro-CT. The coordinate system was established based on the root apex as the origin point (O point). The sample was scanned and divided into several average layers. The distance from each layer to the O point was noted and each layer was nominated a, b, c, d, e, f, and g in turn. The cracks were simulated by three lines in the picture. The red line appeared in the d layer for the first time and the g layer for the last time; the length of the line was noted as “g–d”. The green line appeared in the e layer for the first time and the g layer for the last time; the length of the line was noted as “g–e”. The blue line appeared in the c layer for the first time and the f layer for the last time; the length of the line was noted as “f–c”. The cracked length of each sample was measured.

The measurement of the crack depth is shown in a schematic diagram (**Fig 4**). Each scanned image of the sample was analyzed by NRecon in the y direction (scan l was perpendicular to the tooth’s long axis) and were denominated in sequence. Taking the root apex as the starting point (O point), every image of each tooth was denominated in turn (a, b, c, d, e, f, g) according to the distance to the O point in the y-axis. For instance, the first scanned image of the crack was noted as “a” while the last one was noted as “f”, then the exact length of the crack was obtained using “f–a”. If the direction of the crack was not parallel to the tooth’s long axis, the length of the crack was measured directly on the image by the measuring instrument of NRecon.

### Statistical analysis

SAS 9.2 (SAS software institution, North Carolina, USA) statistics software was used for the statistical data analysis. McNemar’s chi-square test was used for checking the inter-rater consistency between the results of the PR and CBCT diagnosis by the three observers, as well as the sensitivity of the PR and CBCT diagnosis. The diagnostic value of PR and CBCT results across three observers for detecting the cracks depth in teeth was analyzed using receiver operating characteristic curves (ROCs). The level for statistical significance was set at *P* < 0.05.

## Results

The results of CBCT showed that the external consistency ICC with 95% confidence interval (95% CI) of different observers (an endodontic graduate, an endodontist, and a radiologist) was 0.76 (0.64–0.85). The results of PR showed that the external consistency ICC (95% CI) was 0.60 (0.44–0.74). (**Table 1**).

**Table 1:** The agreement of the three observers.

	ICC	95% CI for ICC		F	P
		Lower	Upper		
**CBCT**	0.76	0.64	0.85	10.28	< 0.001
**PR**	0.60	0.44	0.74	5.53	< 0.001

ICC: Intraclass Correlation Coefficient.

In the group of results read by the graduate student, the sensitivity (95% CI) of diagnosis with CBCT and PR were 77.2% (64.8 ~ 89.6) and 9.10% (0.6 ~ 17.6), respectively. In the group of results read by the endodontist, the sensitivity (95% CI) of diagnosis with CBCT was 81.8% (70.4 ~ 93.2) and 11.3% (1.9 ~ 20.7) with PR. In the group of results read by the radiologist, the sensitivity (95% CI) of diagnosis with CBCT and PR were 88.6% (79.2 ~ 98.0) and 11.3% (1.9 ~ 20.7), respectively.

The results of the CBCT and PR were not consistent in diagnosing cracks by the graduate student, endodontist and radiologist. The McNemar test revelaed that this difference was statistically significance (P < 0.001) (**Table 2**).

**Table 2:** Positive and Negative Results of Crack Diagnosis with CBCT and PR of Three Observers (Unit: number).

CBCT	PR					
	Graduate		Endodontist		Radiologist	
	+	-	+	-	+	-
**+**	4	30	5	31	5	34
**-**	0	10	0	8	0	5
**S**	30.00		31.00		34.00	
**P**	< 0.001		< 0.001		< 0.001	

S: standard variance.

The diagnostic value of the PR and CBCT results across the three observers for detecting the depth of cracks in teeth was analyzed using ROC curves (**Table 3 and** **Table** 4).

**Table 3:** Analysis of ROC Curves for CBCT Results across Three Observers.

	AUC	SD	P	95%CI for AUC		Cutoff	Sensitivity
				Lower	Upper		
**Graduate**	0.971	0.028	0.000	0.000	1.000	3.20	0.970
**Endodontist**	1.000	0.000	0.000	0.000	1.000	2.06	1.000
**Radiologist**	1.000	0.000	0.000	0.000	1.000	1.24	1.000

CI, confidence interval; AUC, area under the curve; SD: standard deviation.

**Table 4:** Analysis of ROC Curves for PR Results across Three Observers.

	AUC	SD	P	95%CI for AUC		Cutoff	sensitivity
				Lower	Upper		
**Graduate**	0.819	0.128	0.037	0.000	1.000	6.12	0.750
**Endodontist**	0.995	0.008	0.000	0.000	1.000	6.94	1.000
**Radiologist**	0.995	0.008	0.000	0.000	1.000	6.94	1.000

CI, confidence interval; AUC, area under the curve; SD: standard deviation.

## Discussion

In clinical practice, it is a huge challenge for endodontists to know the depth of a crack in a cracked tooth. Only by confirming the range of the crack, the depth of invasion, the distance to the pulp chamber, and that to the root canal, can an appropriate treatment plan be drawn up, and a long-time prognosis evaluated. Being able to do so may thus improve dentist-patient communication and reduce poor prognoses in cracked teeth. Ultimately, it may obviate the embarrassment of tooth extraction.

The primary idea of the design of this research was firstly based on the different coefficient of thermal expansion of enamel and dentin. Secondly, in the 1970s, Brown proved that enamel will crack when subjected to thousands of rounds of thermal-cycling through 0–50°C [17]. Thirdly, when patients have thermal-cycling eating habits over a long period, cracks may occur in teeth [18], this may be particularly relevant to the eating habits of some Chinese individuals. Therefore, the artificial cracked tooth model was established by immersion in 100°C hot water for 10 min, and then the teeth were quickly transferred into liquid nitrogen (-130°C) for 1 min.

Our results indicate that the sensitivity of diagnosis of cracked teeth in an artificial simulation models with CBCT (77.2%, 81.8%, and 88.6%) was significantly higher than with PR (9.10%, 11.3%, and 11.3%). CBCT was able to detect cracks in the coronal, horizontal, and sagittal plane, but still missed 20–30% of the cracks present in this study. The possible reasons may be: ① crack width. Senem [19] compared the diagnosis of Vertical Root Fractures with three different widths between CBCT and root X-ray photographs. This research showed that the width of the crack could influence the diagnosis of CBCT. ② The difference of CBCT vision and brands. CBCT vision directly affects the voxel size [10], contrast, and resolution. The lower the visual field, the greater the contrast and resolution, and the higher the definition of the picture [20–22]. Hassan [11] et al. concluded that different brands of CBCT may affect the quality of images. The CBCT apparatus in this study was from the German KaVo Company, with a voxel size of 0.25 mm. The smaller the field of view, the higher the image resolution, but the potential for increases in noise is higher as well. Thus, only increasing the dose of CBCT is feasible if images of high resolution are sought. However, this must comply with the principle of using the lowest radiation dose to obtain satisfactory images (as low as reasonably achievable, ALAR) [23]. Therefore, such a dose increase is infeasible in clinical practice.

Based on our study, the depth of the crack has a strong influence on the diagnosis of a cracked tooth, not only with CBCT, but also with PR. For CBCT diagnosis, the cutoff value for the graduate, endodontist, and radiologist was 3.20 mm, 2.06 mm, and 1.24 mm, respectively. For PR diagnosis, the cutoff value for the graduate, endodontist, and radiologist was 6.12 mm, 6.94 mm, and 6.94 mm, respectively. Therefore, the cutoff value for the endodontist to diagnose with CBCT was 2.06 mm, and the cutoff value for the radiologist was 1.24 mm, while for the endodontic graduate student, it was 3.20 mm. On the principle of early diagnosis, it would be better that radiologists diagnose cracked teeth with CBCT, as they can detect a minimum depth of 1.24 mm.

The ROC curves in our results require some further explanation. Generally speaking, when micro-CT cannot detect a crack, neither can CBCT or PR. We did not include the teeth without any cracks at the beginning of this study, and thus could not make any ROC curve using data from micro-CT. Only data from CBCT and PR could make respective ROC curves.

Moreover, the experience of different observers could be one of the factors that can influence the diagnosis of tooth cracks. For the graduate student, the sensitivity of diagnosis with CBCT was 77.2%, but with PR it was 9.10%. For the endodontist, the sensitivity of diagnosis with CBCT was 81.8%, but 11.3% with PR. For the radiologist, the sensitivity of diagnosis with CBCT was 88.6%, and with PR it was 11.3%. Therefore, when the three observers, that is, an inexperienced endodontic graduate student, an experienced endodontist, and a certified radiologist were challenged with subtle cracks, only radiologists had the penetrating insight required in detecting medically abnormal images; therefore, we recommend that it will be better if cracked teeth are diagnosed by radiologists, especially for facilitating early diagnosis. Endodontic graduate students and endodontists should also be trained in diagnostic oral radiology, especially to their reading of PR and CBCT images with cracks.

Last but not least, the measurement could be another factor that can influence the diagnosis of tooth cracks. The results of CBCT showed that the external consistency ICC of the three observers was 0.76. For PR results the external consistency ICC was 0.60, indicating that CBCT diagnostic consistency was high, and PR diagnostic consistency was moderate among the three individuals. Compared with CBCT diagnosis, PR may have a higher false negative rate, since the ICC was lower. Therefore, it is recommended that cracked teeth are diagnosed using CBCT, especially for early diagnosis.

Generally speaking, the area under the curve (AUC) can be very close to 1.0, and the closer the better, but cannot actually be 1.0 itself. In our results in Table 3, the AUCs for the endodontist and radiologist were very close to 1.0. This was because nearly all cracks were detected by the endodontist and radiologist with CBCT, and the sensitivity was close 1.00, which means the endodontist and radiologist had a high diagnostic accuracy for crack diagnosis with CBCT.

There were a few limitations in our study. Firstly, this study was conducted on an artificial simulation models of cracked teeth. The samples were processed *in vitro*, and there were no alveolar bones, periodontal membranes, dental pulp tissue, or adjacent teeth. Regardless of these factors, the quality of CBCT and PR images may be higher than those obtained in actual clinical conditions. In addition, the pixel size with CBCT was 0.25 mm × 0.25 mm. Secondly, this research is a preliminary study with a relatively small sample size of 44 teeth. Thirdly, it lacks validation with clinical data. Researchers need to design more *in vitro* and clinical trials investigating cracked teeth, as well as develop more valuable instruments and equipment for diagnosis. Further studies will be required to confirm the findings of the present study.

## Supporting Information

S1 Table Primary data of the study. (DOC)

S2 Table Primary data of the study, continued. (XLSX)

## References

[1] Geurtsen W, Schwarze T, Gunay H. Diagnosis, therapy, and prevention of the cracked tooth syndrome[J]. Quintessence international (Berlin, Germany: 1985). 2003;34(6):409–17. 12859085

[2] Lubisich EB, Hilton TJ, Ferracane J, Northwest P. Cracked teeth: a review of the literature[J]. Journal of esthetic and restorative dentistry: official publication of the American Academy of Esthetic Dentistry. 2010;22(3):158–67. 10.1111/j.1708-8240.2010.00330.x PMC3870147 20590967

[3] Mathew S, Thangavel B, Mathew CA, Kailasam S, Kumaravadivel K, Das A. Diagnosis of cracked tooth syndrome[J]. Journal of pharmacy & bioallied sciences. 2012;4(Suppl 2):S242–4. 23066261 10.4103/0975-7406.100219 PMC3467890

[4] Kim SY, Kim SH, Cho SB, Lee GO, Yang SE. Different treatment protocols for different pulpal and periapical diagnoses of 72 cracked teeth[J]. Journal of endodontics. 2013;39(4):449–52. 10.1016/j.joen.2012.11.052 23522534

[5] Michaelson PL. A novel treatment for propagated crown fractures[J]. Journal of endodontics. 2015;41(1):130–4. 10.1016/j.joen.2014.06.021 25132190

[6] Christensen GJ. When is a full-crown restoration indicated[J]. Journal of the American Dental Association. 2007;138(1):101–3. 17197409 10.14219/jada.archive.2007.0028

[7] Opdam NJ, Roeters JJ, Loomans BA, Bronkhorst EM. Seven-year clinical evaluation of painful cracked teeth restored with a direct composite restoration[J]. Journal of endodontics. 2008;34(7):808–11. 10.1016/j.joen.2008.04.011 18570984

[8] Kim S. Endodontic application of cone-beam computed tomography in South Korea[J]. Journal of endodontics. 2012;38(2):153–7. 10.1016/j.joen.2011.09.008 22244628

[9] Andreasen JO, Andersson L, Andreasen FM. Textbook and Color Atlas of Dental Trauma. Beijing People's Medical Publishing House 2012.

[10] Hassan B, Metska ME, Ozok AR, van der Stelt P, Wesselink PR. Detection of vertical root fractures in endodontically treated teeth by a cone beam computed tomography scan[J]. Journal of endodontics. 2009;35(5):719–22. 10.1016/j.joen.2009.01.022 19410091

[11] Hassan B, Metska ME, Ozok AR, van der Stelt P, Wesselink PR. Comparison of five cone beam computed tomography systems for the detection of vertical root fractures[J]. Journal of endodontics. 2010;36(1):126–9. 10.1016/j.joen.2009.09.013 20003950

[12] Melo SL, Bortoluzzi EA, Abreu M Jr., Correa LR, Correa M. Diagnostic ability of a cone-beam computed tomography scan to assess longitudinal root fractures in prosthetically treated teeth[J]. Journal of endodontics. 2010;36(11):1879–82. 10.1016/j.joen.2010.08.025 20951305

[13] May JJ, Cohenca N, Peters OA. Contemporary management of horizontal root fractures to the permanent dentition: diagnosis—radiologic assessment to include cone-beam computed tomography[J]. Pediatric dentistry. 2013;35(2):120–4. 23635979

[14] Scarfe WC, Levin MD, Gane D. Review Article Use of Cone Beam Computed Tomography in Endodontics[J]. International Journal of Dentistry. 2009;2009:1–20. 10.1155/2009/634567 PMC2850139 20379362

[15] Todd R. Cone beam computed tomography updated technology for endodontic diagnosis[J]. Dental clinics of North America. 2014;58(3):523–43. 10.1016/j.cden.2014.03.003 24993922

[16] Venskutonis T, Plotino G, Juodzbalys G, Mickeviciene L. The importance of cone-beam computed tomography in the management of endodontic problems: a review ofthe literature[J]. Journal of endodontics. 2014;40(12):1895–901. 10.1016/j.joen.2014.05.009 25287321

[17] Lloyd BA, McGinley MB, Brown WS. Thermal stress in teeth. Journal of dental research 1978,57(4):571–582. 280571 10.1177/00220345780570040701

[18] Berman LH, Kuttler S. Fracture necrosis: diagnosis, prognosis assessment, and treatment recommendations. Journal of endodontics 2010,36(3):442–446. 10.1016/j.joen.2009.12.018 20171360

[19] Özer Senem Yiğit. Detection of Vertical Root Fractures of Different Thicknesses in Endodontically Enlarged Teeth by Cone Beam Computed Tomography versus, Digital Radiography[J]. Journal of Endodontics, 2010, 36(7):1245–9. 10.1016/j.joen.2010.03.021 20630309

[20] Katsumata A, Hirukawa A, Okumura S, Naitoh M, Fujishita M, Ariji E, et al Effects of image artifacts on gray-value density in limited-volume cone-beam computerized tomography[J]. Oral surgery, oral medicine, oral pathology, oral radiology, andendodontics. 2007;104(6):829–36. 10.1016/j.tripleo.2006.12.005 17448704

[21] Katsumata A, Hirukawa A, Okumura S, Naitoh M, Fujishita M, Ariji E, et al Relationship between density variability and imaging volume size in cone-beam computerized tomographic scanning of the maxillofacial region: an in vitro study[J]. Oral surgery, oral medicine, oral pathology, oral radiology, and endodontics. 2009;107(3):420–5. 10.1016/j.tripleo.2008.05.049 18715805

[22] Liang X, Jacobs R, Hassan B, Li L, Pauwels R, Corpas L, et al A comparative evaluation of Cone Beam Computed Tomography (CBCT) and Multi-Slice CT (MSCT) Part I. On subjective image quality[J]. European journal of radiology. 2010;75(2):265–9. 10.1016/j.ejrad.2009.03.042 19410409

[23] Farman AG. ALARA still applies[J]. Oral surgery, oral medicine, oral pathology, oral radiology, and endodontics. 2005;100(4):395–7. 10.1016/j.tripleo.2005.05.055 16182157

