# Supplementary material for: The Extent of the Crack on Artificial Simulation Models with CBCT and Periapical Radiography
Source: PLoS One. 2017 Jan 4;12(1):e0169150. doi: 10.1371/journal.pone.0169150 (PMC5215233; doi:10.1371/journal.pone.0169150)
Supplement: S1 Table — (DOC) [file pone.0169150.s001.doc]

**Table.** Depth of Crack of 44 Cracked Teeth on Micro-CT (Unit: mm)

| Numbers | 1-11 12-22 23-33 34-44 |
| --- | --- |
| 1 4.303 3.600 2.099 4.276  2 2.019 5.332 3.426 3.958  3 3.480 9.856 8.182 3.391  4 5.632 6.300 4.516 3.685  5 4.356 0.478 13.972 8.235  6 0.644 8.723 3.400 4.568  7 4.011 3.540 3.931 4.144  8 4.171 3.400 6.880 5.860  9 2.975 4.648 1.912 4.648  10 0.425 4.700 5.153 5.950  11 0.133 7.000 1.833 0.356 | |
